# Novel Poly(*ɛ*-caprolactone)/Graphene Scaffolds for Bone Cancer Treatment and Bone Regeneration

**DOI:** 10.1089/3dp.2020.0051

**Published:** 2020-10-15

**Authors:** Yanhao Hou, Weiguang Wang, Paulo Bártolo

**Affiliations:** Department of Mechanical, Aerospace and Civil Engineering, School of Engineering, Faculty of Science and Engineering, The University of Manchester, Manchester, United Kingdom.

**Keywords:** biomanufacturing, cancer, graphene, poly(*ɛ*-caprolactone), scaffolds, tissue engineering

## Abstract

Scaffold-based bone tissue engineering is the most relevant approach for critical-sized bone defects. It is based on the use of three-dimensional substrates to provide the appropriate biomechanical environment for bone regeneration. Despite some successful results previously reported, scaffolds were never designed for disease treatment applications. This article proposes a novel dual-functional scaffold for cancer applications, comprising both treatment and regeneration functions. These functions are achieved by combining a biocompatible and biodegradable polymer and graphene. Results indicate that high concentrations of graphene enhance the mechanical properties of the scaffolds, also increasing the inhibition on cancer cell viability and proliferation.

## Introduction

Primary bone cancer is a kind of sarcoma, with around 700,000 new cases being reported per year worldwide.^1,2^ According to a report from the National Cancer Intelligence Network (NCIN), between 1985 and 2009, an average of 380 new primary bone cancer cases were diagnosed per year in England.^3^ Due to its low incidence, delayed diagnosed and treatment are common, leading to a high mortality (worldwide more than 220,000 patients died per year after treatment and the survival rate of these patients is around 68%).^1^ Depending on the type and extension of cancer cells, the main clinical strategies to treat bone cancer comprise chemotherapy, radiotherapy, targeted cancer drugs, surgery, and a combination of these methods (Table 1).^3–5^ However, these therapeutic strategies present several physical and psychological side effects (pain, constipation, diarrhea, allergies, hair loss, low immunity, superinfection, multiple surgeries, and amputations).^6,7^ Due to these limitations, new and more effective strategies are still required.

**Table 1.** Common Cancer Types, Corresponding Treatment, and Their Limitations

Cancer type	Corresponding treatment	Limitations
Osteosarcoma	Chemotherapy	Hair loss, diarrhea or constipation, lower resistance to infections, easily bleeding and bruising
Ewing's sarcoma	Chemotherapy, radiotherapy	Diarrhea or constipation, lower resistance to infections, easily bleeding and bruising, skin red or dark, hair loss, and pain
Chondrosarcoma	Radiotherapy, surgery	Skin red or dark, hair loss and pain, amputation
Chordoma	Radiotherapy	Skin red or dark, hair loss, and pain

**FIG. 1.**
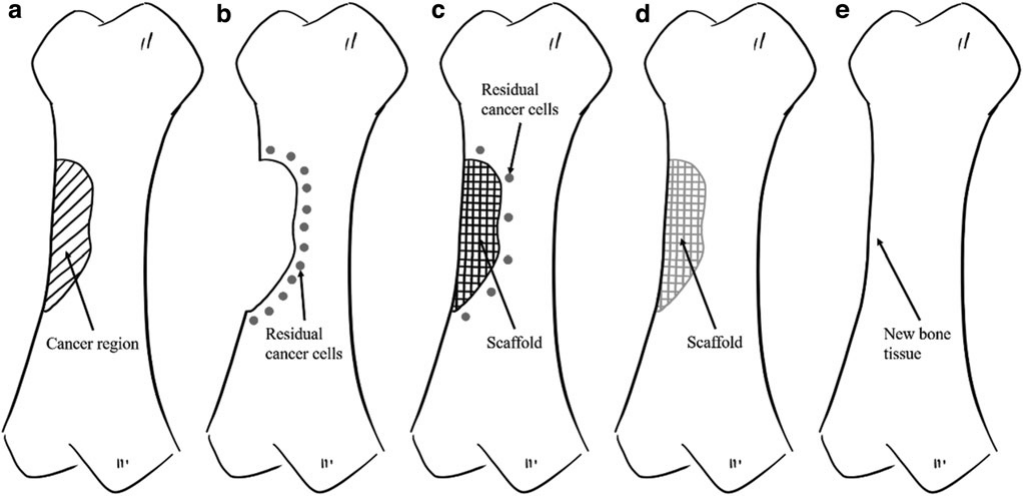
Treatment and repair of bone cancer **(a)** Bone with a cancer region, **(b)** Bone with possible residual cancer cells after the bone cancer region being removed, **(c)** Implanted dual-function scaffold. Treatment phase: Initial layers of the scaffold containing a cytotoxic material will induce the death of the possible remaining cancer cells in the surrounding tissue, **(d)** Regeneration phase: After a certain period of time, the layers responsible for the treatment phase disappear (biodegradable) and layers of biocompatible materials will start recruiting healthy cells, promoting cell attachment, proliferation, and differentiation, and inducing new bone formation. **(e)** Bone with regenerated tissue.

Based on our extensive experience in designing three-dimensional (3D) bone scaffolds for both *in vitro* and *in vivo* applications,^8–16^ we decided to start a new research program aiming at developing a novel concept of dual-function 3D scaffolds for bone cancer applications to (Fig. 1):

- Induce the death of cancer cells (phase 1)—treatment function.
- Recruit healthy cells, inducing new bone formation (phase 2)—regeneration function.

Novel dual-function scaffolds are mainly made with a biodegradable and biocompatible polymer. This polymer is poly(*ɛ*-caprolactone) (PCL), a semi-crystalline aliphatic polymer that has been successfully used by our group for bone tissue engineering scaffolds. To design effective PCL-based scaffolds, we investigated the degradation kinetics of such scaffolds as a function of scaffold topology,^17,18^ the effect of processing conditions on the morphological development/microstructure formation during the printing process,^19,20^ and surface modification strategies to improve cell attachment, proliferation, and differentiation.^16,21^ Due to the piezoelectric and reverse piezoelectric properties of bone, our group also developed strategies to induce electroconductive properties on PCL-based scaffolds by adding a low concentration of conductive materials such as graphene.^9,12,16^

Previously reported studies demonstrated that graphene, 2D single-atom thick sheets of carbon atoms bound in hexagonal lattice structures, shows high affinity to glioblastoma cells leading to the adhesion of graphene to the cell's body and, consequently, cell membrane damage. Other studies also reported different levels of cytotoxicity both *in vitro* and *in vivo* depending on the graphene form (pristine graphene, graphene oxide, and reduced graphene oxide), showing that toxic effects are concentration-, time- and shape-dependent.^10,22,23^ Graphene has been reported to induce oxidative stresses and cell function assays, and confocal microscopy images indicated that reactive oxygen species induced by the carbon atoms on the edge of graphene can damage DNA.^24–26^ Moreover, authors also reported that the few-layer graphene dispersions have a specific killing ability on monocytes.^27^ Graphene also showed an effective ability to inhibit the migration and invasion of human breast and prostate cancer cells, as well as mouse melanoma cells.^28^

**FIG. 2.**
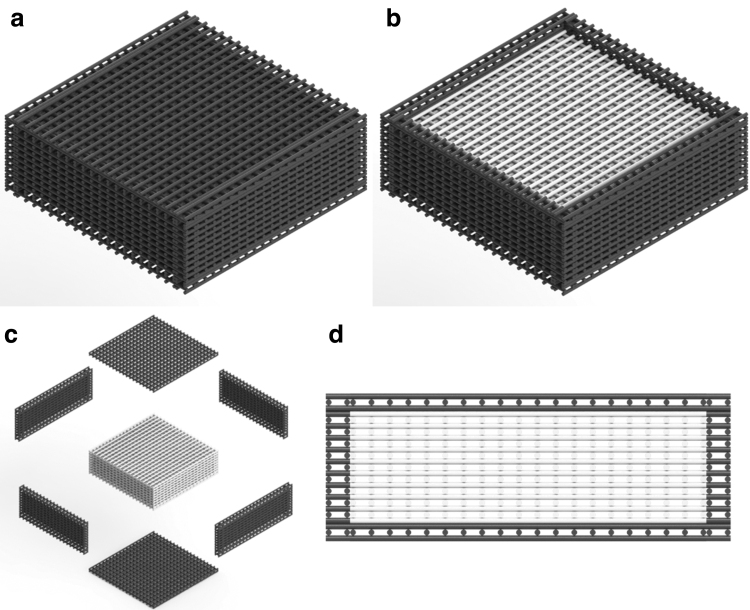
Dual-functional scaffolds **(a)** Overall view, **(b)** Overall view (*without top*), **(c)** Exploded view showing outer layers responsible for the treatment function and inner layers responsible for the regeneration function, **(d)** Side view (*without one side*).

The potential cytotoxicity of high graphene contents on a PCL matrix are explored in this article to create the first layer of the novel dual-function scaffolds (Fig. 2). Graphene, uniformly dispersed in the PCL fibers,^14^ will be gradually released, inducing the apoptosis of the remaining cancer cells. This effect can be controlled by the graphene content, and both crystallinity and crystal orientation of PCL in the printed fibers, which strongly depend on processing conditions. After the degradation of these initial layers that are responsible for the treatment function of the scaffold, PCL layers (core part of the scaffold) will be revealed. These layers must be able to provide the necessary biomechanical environmental conditions to sustain the recruitment of healthy cells (bone cells or stem cells) and cell attachment, proliferation, and differentiation (regeneration function).

## Materials and Methods

### Scaffolds fabrication

Graphene nanosheets were synthesized from graphite by water-assisted liquid-phase exfoliation, as reported earlier.^9^ The obtained graphene nanosheets were mixed with PCL pellets (CAPA 6500; Perstorp, United Kingdom) through a melt-blending process by using different graphene concentrations (5 wt.%, 7 wt.%, and 9 wt.%). The PCL pellets and graphene nanosheets were heated to 150°C and mixed together in a crucible for 30 min. The material was stirred for 30 min to homogeneously disperse the graphene. After cooling down for 3 h, the mixed materials were then cut into pellets and prepared for printing.

Scaffolds were produced by a screw-based additive manufacturing system 3DDiscovery™ Evolution Bench-top (screw length 45 mm, pitch 5.15 mm, diameter 10 mm) (regenHU, Switzerland), which allows to print fibers with a uniform dispersion of graphene as previously reported by micro-Raman spectroscopy.^14^ The following design and processing parameters were considered:

- Design parameters:
- Laydown pattern: 0°/90°.
- Square pores.
- Pore size: 350 μm.
- Filament diameter: 330 μm.
- Slice thickness: 270 μm.
- Processing parameters:
- Melt temperature: 90°C.
- Screw rotation velocity: 8 rpm.
- Deposit velocity: 12 mm/s.
- Pressure: 6 bar.

### Thermogravimetric analysis

Thermogravimetric analysis (TGA) was used to determine the exact amount of graphene present in the scaffold. Tests were performed by using the Thermal Analysis Q500 analyzer (TA Instrument) with PCL scaffolds and graphene-loaded scaffolds. Scans were performed in an air atmosphere with an airflow of 60 mL/min and temperature ranging from 35°C to 560°C at a 10°C/min ranging rate. TGA tests were performed by using samples with around 1 g of weight placed in platinum pans. The weight change of the samples was monitored by using the TA Universal Analysis 2000 software (TA Instrument).

### Morphological evaluation

Morphological characterization of the scaffolds was performed through scanning electron microscopy (SEM). The Hitachi S3000N system (Hitachi, United Kingdom) was used to capture images of both the top surface and cross-section of the scaffolds, using an accelerating voltage of 10 kV. Before image capturing, the scaffolds were cut into 4 mm blocks and coated with a thin layer of metal (10 nm gold) by using the EMITECH K550X sputter coater (Quorum Technologies, United Kingdom). The obtained images were processed by ImageJ (NIH).

### Mechanical evaluation

Uni-axial compression tests were conducted on the INSTRON X testing system (High Wycombe, United Kingdom) with a 100 N load cell. The scaffolds were cut into blocks of 3 mm of width, 3 mm of length, and 4 mm of height. Compression tests were performed in dry state with the strain ranging from 0 to 0.3 mm/mm (30%) and a displacement rate of 0.5 mm/min. Force *F* and corresponding displacement Δ*h* were measured by sensors while the samples were compressed, and they were then transformed into stress *σ* and strain *ɛ* values. The compressive modulus and compressive strength of the scaffolds were calculated and plotted by Origin (OriginLab).

### Biological studies

*In vitro* biological studies were conducted by using both human adipose-derived stem cells (hADSCs) (Invitrogen) and sarcoma osteogenic (Saos-2) cells (ATCC). Cells were cultured in the corresponding medium (MesenPRO RS™ basal medium [Thermo Fisher Scientific] for hADSCs and McCoy's 5A Medium [Sigma-Aldrich, United Kingdom] for Saos-2 cells) in T75 cell culture flasks (Sigma-Aldrich, United Kingdom) at standard condition (37°C, 5% CO_2_, concentration and 95% humidity) in a New Brunswick^®^ Galaxy 170 R incubator (Eppendorf). Cells were harvested at approximate 80% confluency with 0.05% trypsin-ethylenediaminetetraacetic acid (Invitrogen) before cell seeding. Cells ranging from passage 6 to 8 were considered for biological evaluation.

Before cell seeding, scaffolds were sterilized in 80% ethanol (Thermo Fisher Scientific) for 4 h, transferred to a 24-well plate, and rinsed three times with Dulbecco's phosphate buffered saline (Thermo Fisher Scientific). Fifty-thousand cells (counted by Cellometer Auto 1000 Bright Field Cell Counter [Nexcelom Bioscience]) in 0.8 mL corresponding medium were seeded on each scaffold sample and empty well (control group).

Cell viability was evaluated at 1, 3, and 7 days after cell seeding by using the Alamar Blue assay, which can quantitatively monitor the metabolism and cytotoxicity of the cells.^29^ At each particular time point, 0.8 mL of medium containing 0.001% Alamar Blue (Sigma-Aldrich, United Kingdom) was added to each well and incubated for 4 h. Then, 150 μL fluid from each well was transferred into a 96-well plate and the fluorescence intensity was measured by a Multi-Detection Microplate Reader Synergy HT (BioTec) (excitation wavelength of 540 nm and emission wavelength of 590 nm).

## Results and Discussion

### Thermogravimetric analysis

**FIG. 3.**
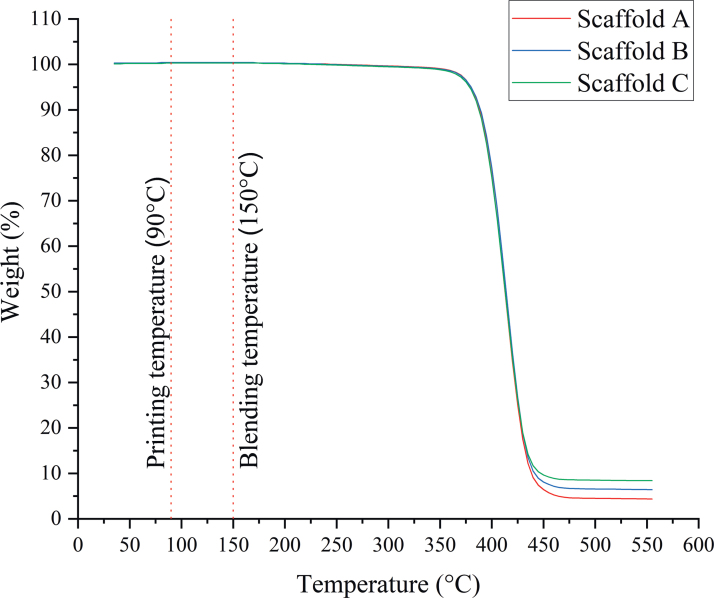
Temperature-weight curves for the scaffolds with different graphene contents. Color images are available online.

Table 2 presents the results of the final content of graphene on the composite scaffolds. Results show that graphene was effectively incorporated into the scaffold without significant losses during both the melt-blending and printing processes. Figure 3 also shows that no degradation event occurs during both composite preparation and printing processes, confirming that both material preparation and scaffold fabrication do not induce any transformation on the materials.

**Table 2.** Graphene Content on Composite Scaffolds Determined by Thermogravimetric Analysis

Scaffolds	Initial graphene loading (wt.%)	Final graphene content (wt.%)	Scaffolds reference
PCL	—	—	PCL
PCL/graphene	5	4.37	A
	7	6.41	B
	9	8.40	C

PCL, poly(*ɛ*-caprolactone).

### Morphological evaluation

**FIG. 4.**
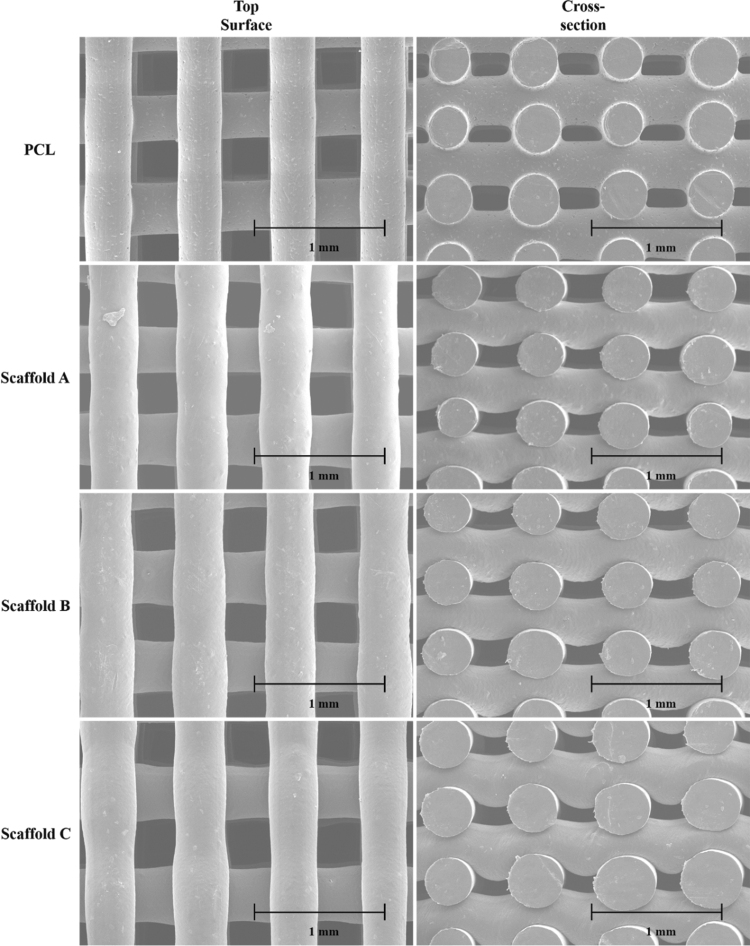
*Top* surface and cross-section SEM images of printed scaffolds. SEM, scanning electron microscopy.

The SEM images of both the top surface and cross-section of the scaffolds are presented in Figure 4. Printed scaffolds present an average filament diameter of 361.06 ± 22.71 μm (the designed value was 330 μm), regular square pores with an average pore size of 340.05 ± 20.91 μm in the vertical direction (top surface, the designed value was 350 μm), and 143.29 ± 30.35 μm in the horizontal direction (cross-section, the designed value was 210 μm). Filament diameter increases from 336.36 ± 8.41 μm (PCL scaffold) to 363.81 ± 14.76 μm (Scaffold A) and 380.87 ± 16.54 (Scaffold B) and then decreases to 363.18 ± 23.10 μm (Scaffold C) by increasing the graphene content. Pore size in the vertical direction decreases from 363.71 ± 11.58 μm (PCL scaffold) to 321.68 ± 14.75 μm (Scaffold A) and then increases to 332.45 ± 17.16 μm (Scaffold B) and 335.46 ± 14.98 μm (Scaffold C) by increasing the graphene content. Pore size in the horizontal direction increases from 142.66 ± 31.44 μm (PCL scaffold) to 164.41 ± 28.71 μm (Scaffold A) and then decreases to 147.26 ± 25.94 μm (Scaffold B) and 118.84 ± 17.13 μm (Scaffold C) by increasing the graphene content. These variations are due to rheological (viscosity, shear-thinning, and viscoelastic properties) effects associated with the different material composition. Based on this information, it would be possible, in the future, to adjust processing parameters to produce scaffolds with similar topological characteristics.

### Mechanical evaluation

**FIG. 5.**
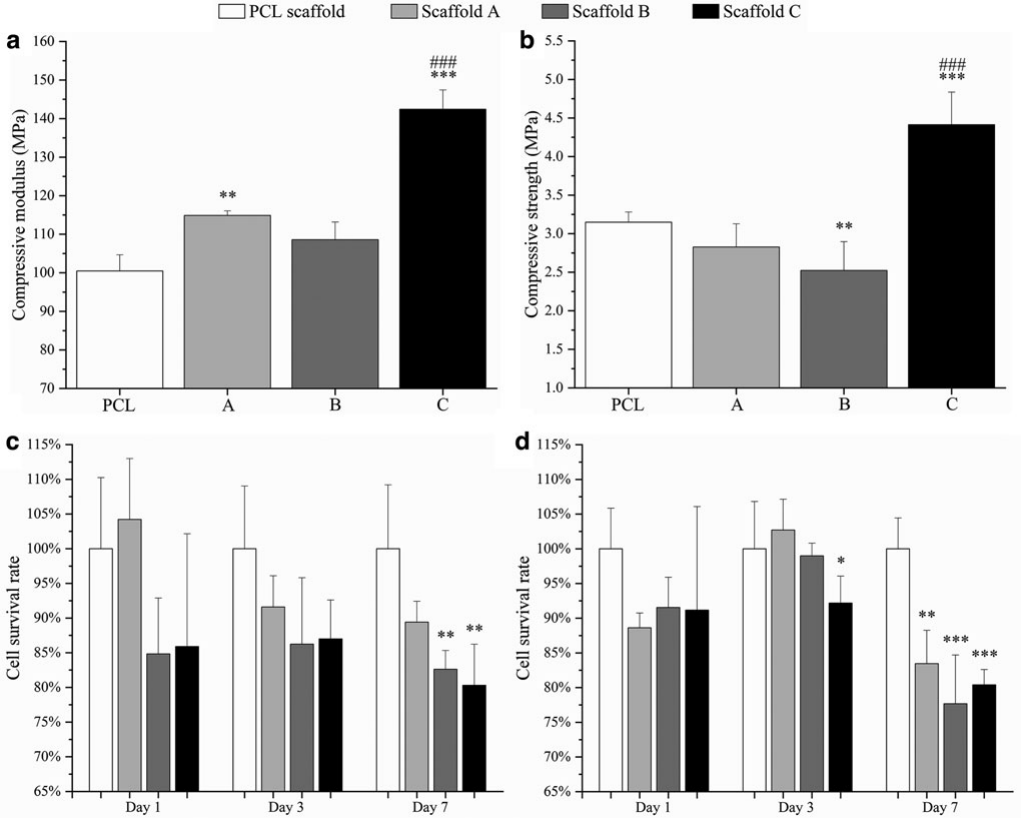
Mechanical properties of scaffolds with different graphene contents. **(a)** Compressive modulus; **(b)** Compressive strength. Percentage of survival cells after 1, 3, and 7 days after cell seeding with PCL scaffold as the reference. **(c)** hADSCs; **(d)** Saos-2 cells. One-way analyses of variance (one-way ANOVA) with Tukey test were carried out by Origin due to the only variable being the concentration of graphene fillers. The significance levels were set at *p < 0.05, **p < 0.01, ***p < 0.001, compared with the control (PCL), and ^###^*p* < 0.001 regarding different graphene concentrations. ANOVA, analysis of variance; hADSCs, human adipose-derived stem cells; PCL, poly(*ɛ*-caprolactone); Saos-2, sarcoma osteogenic.

Figure 5a and b show compressive modulus and compressive strength for both PCL and PCL/graphene scaffolds. As observed, the addition of graphene fillers increased the compressive modulus from 100.46 ± 4.22 MPa (PCL scaffolds) to 142.41 ± 5.01 MPa (Scaffold C). The incorporation of graphene fillers also enhanced the compressive strength from 3.15 ± 0.13 MPa (PCL scaffolds) to 4.41 ± 0.42 MPa (Scaffold C). Results also show that the fabricated scaffolds have the same order of magnitude of mechanical properties as human trabecular bone. Depending on gender and age, trabecular bone presents compressive modulus ranging from 50 to 1500 MPa with the mean value of 194 MPa, and the compressive strength ranges from 1 to 30 MPa with the mean value of 3.55 MPa.^30–33^

### Biological studies

Biological evaluation results are presented in Figure 5c and d, showing the fluorescence intensity values at different time points for different scaffolds. The fluorescence intensity is proportional to the number of metabolically active cells, indicating the cell survival rate. As observed, after days 1 and 3 of cell seeding, although the scaffolds containing graphene exhibited a negative effect on cell viability and proliferation, no statistical differences were observed between scaffold A, scaffold B, and PCL scaffold. Only scaffold C showed a statistically lower survival rate of Saos-2 cells. However, after 7 days of cell seeding, scaffolds B and C presented statistically lower fluorescence intensity on both hADSCs and Saos-2 cells than the PCL scaffold, indicating lower cell viability and proliferation rate. Scaffold A also showed a statistically lower survival rate on Saos-2 cells. Results also indicate that all scaffolds presented a relatively lower cell survival rate on Saos-2 cells than hADSCs (83.5% in the case of Saos-2 cells and 89.4% in the case of hADSCs on scaffold A; 77.7% in the case of Saos-2 cells and 82.6% in the case of hADSCs on scaffold B; 80.4% in the case of Saos-2 cells and 80.3% in the case of hADSCs on scaffold C).

These results show that the addition of graphene fillers can reduce the survival rate of both hADSCs and Saos-2 cells, and the PCL/graphene scaffolds exhibit a greater inhibition ability on Saos-2 cells than on hADSCs. Besides, cells can grow and proliferate well on PCL scaffolds between days 1 and 7, showing no significant inhibition effect.

## Conclusions and Future Perspectives

This article proposes for the first time a novel dual-functional 3D scaffold for bone cancer treatment and regeneration post-treatment. These scaffolds comprise external layers made of PCL and high contents of graphene and internal PCL layers. Results show the inhibitory effect of graphene, particularly on Saos-2 cells, and the high load of cell attachment and proliferation on PCL. The addition of graphene also contributes to increasing the compressive modulus and compressive strength, thus making it possible to achieve values similar to the human trabecular bone. This is particularly relevant for the initial stages where the scaffold must be able to support the loads in the host environment. All scaffolds were produced by using the same processing conditions; the consequences of rheological changes due to modifications on the material composition, differences in terms of filament diameters and pore size between printed scaffolds and designed ones were observed. These will be solved in the future by adjusting the processing conditions depending on printability and rheological characterization of the material. Besides, a wider range of materials, including graphene oxide and selenium, will be further investigated. Surface modification will also be considered to allow the incorporation of biomolecules or targeted cancer treatment drugs into the scaffolds. In terms of biological performance, longer *in vitro* studies and other assays [e.g., 3-(4,5-dimethylthiazol-2-yl)-2,5-diphenyltetrazolium bromide (MTT assay) and lactate dehydrogenase (LDH assay)] will be considered before the *in vivo* study. Further, long-term *in vitro* and *in vivo* degradation tests will also be performed. In general, results seem to indicate that the proposed dual-functional scaffold can be a valid solution for bone cancer applications, without the secondary effects of current clinical approaches focusing on treatment and not on both treatment and regeneration.

## References

[1] Franchi A Epidemiology and classification of bone tumors. Clin Cases Miner Bone Metab 2012;9:92–95 23087718 PMC3476517

[2] Kumar N, Gupta B Global incidence of primary malignant bone tumors. Curr Orthop Pract 2016;27:530–534

[3] Gerrand C, Athanasou N, Brennan B, *et al.* On behalf of the British Sarcoma, G. UK guidelines for the management of bone sarcomas. Clin Sarcoma Res 2016;6:7 27148438 10.1186/s13569-016-0047-1 PMC4855334

[4] Canal C, Fontelo R, Hamouda I, *et al.* Plasma-induced selectivity in bone cancer cells death. Free Radic Biol Med 2017;110:72–80 28571751 10.1016/j.freeradbiomed.2017.05.023

[5] Grimer R, Athanasou N, Gerrand C, et al. UK guidelines for the management of bone sarcomas. Sarcoma 2010;2010;317462 10.1155/2010/317462 PMC3022187 21253474

[6] van Driel M, van Leeuwen JPTM Cancer and bone: A complex complex. Arch Biochem Biophys 2014;561:159–166 25046842 10.1016/j.abb.2014.07.013

[7] Stolzoff M, Webster TJ Reducing bone cancer cell functions using selenium nanocomposites. J Biomed Mater Res A 2016;104:476–482 26454004 10.1002/jbm.a.35583

[8] Daskalakis E, Aslan E, Liu F, et al. Composite scaffolds for large bone defects. In: Almeida HA, Vasco JC, eds. Proceedings of Progress in Digital and Physical Manufacturing. Cham, Switzerland: Springer International Publishing, 2020; pp. 250–257

[9] Wang WG, Huang BY, Byun JJ, *et al.* Assessment of PCL/carbon material scaffolds for bone regeneration. J Mech Behav Biomed Mater 2019;93:52–60 30769234 10.1016/j.jmbbm.2019.01.020

[10] Caetano G, Wang WG, Murashima A, *et al.* Tissue constructs with human adipose-derived mesenchymal stem cells to treat bone defects in rats. Materials 2019;12:2268 10.3390/ma12142268 PMC6679084 31311087

[11] Huang B, Vyas C, Roberts I, *et al.* Fabrication and characterisation of 3D printed MWCNT composite porous scaffolds for bone regeneration. Mater Sci Eng C Mater Biol Appl 2019;98:266–278 30813027 10.1016/j.msec.2018.12.100

[12] Wang W, Junior JRP, Nalesso PRL, *et al.* Engineered 3D printed poly(ɛ-caprolactone)/graphene scaffolds for bone tissue engineering. Mater Sci Eng C Mater Biol Appl 2019;100:759–770 30948113 10.1016/j.msec.2019.03.047

[13] Daskalakis E, Liu F, Acar AA, *et al.* Polymer-ceramic bone bricks for tissue engineering. Transact Addit Manuf Meets Med 2019;1:1–2

[14] Caetano GF, Wang W, Chiang W-H, *et al.* 3D-printed poly(ɛ-caprolactone)/graphene scaffolds activated with P1-latex protein for bone regeneration. 3d Print Addit Manuf 2018;5:127–137 10.1089/3dp.2018.0012.correx PMC6714346 31479503

[15] Wang W, Caetano GF, Chiang W-H, *et al.* Morphological, mechanical and biological assessment of PCL/pristine graphene scaffolds for bone regeneration. Int J Bioprinting 2016;2:95–104

[16] Wang WG, Caetano G, Ambler WS, *et al.* Enhancing the hydrophilicity and cell attachment of 3D printed PCL/graphene scaffolds for bone tissue engineering. Materials 2016;9:992 10.3390/ma9120992 PMC5456956 28774112

[17] Domingos M, Chiellini F, Cometa S, *et al.* Evaluation of in vitro degradation of PCL scaffolds fabricated via BioExtrusion. Part 1: Influence of the degradation environment. Virtual Phys Prototyp 2010;5:65–73

[18] Domingos M, Chiellini F, Cometa S, *et al.* Evaluation of in vitro degradation of PCL scaffolds fabricated via BioExtrusion—Part 2: Influence of pore size and geometry. Virtual Phys Prototyping 2011;6:157–165

[19] Liu F, Vyas C, Poologasundarampillai G, *et al.* Structural evolution of PCL during melt extrusion 3D printing. Macromol Mater Eng 2018;303:1700494

[20] Liu F, Vyas C, Poologasundarampillai G, *et al.* Process-driven microstructure control in melt-extrusion-based 3D printing for tailorable mechanical properties in a polycaprolactone filament. Macromol Mater Eng 2018;303:1800173

[21] LiuF, Wang W, Mirihanage W, *et al.* A plasma-assisted bioextrusion system for tissue engineering. Cirp Ann Manuf Technol 2018;67:229–232

[22] Lammel T, Boisseaux P, Fernandez-Cruz ML, *et al.* Internalization and cytotoxicity of graphene oxide and carboxyl graphene nanoplatelets in the human hepatocellular carcinoma cell line Hep G2. Part Fibre Toxicol 2013;10:27 23849434 10.1186/1743-8977-10-27 PMC3734190

[23] Zhang Y, Ali SF, Dervishi E, *et al.* Cytotoxicity effects of graphene and single-wall carbon nanotubes in neural phaeochromocytoma-derived PC12 cells. ACS Nano 2010;4:3181–3186 20481456 10.1021/nn1007176

[24] Jarosz A, Skoda M, Dudek I, *et al.* Oxidative stress and mitochondrial activation as the main mechanisms underlying graphene toxicity against human cancer cells. Oxid Med Cell Longev 2016;2016:5851035 26649139 10.1155/2016/5851035 PMC4662972

[25] Sasidharan A, Panchakarla LS, Chandran P, *et al.* Differential nano-bio interactions and toxicity effects of pristine versus functionalized graphene. Nanoscale 2011;3:2461–2464 21562671 10.1039/c1nr10172b

[26] Kumar S, Chatterjee K Comprehensive review on the use of graphene-based substrates for regenerative medicine and biomedical devices. ACS Appl Mater Interfaces 2016;8:26431–26457 27662057 10.1021/acsami.6b09801

[27] Russier J, León V, Orecchioni M, *et al.* Few-layer graphene kills selectively tumor cells from myelomonocytic leukemia patients. Angew Chem Int Ed Engl 2017;56:3014–3019 28156035 10.1002/anie.201700078

[28] Zhou H, Zhang B, Zheng J, *et al.* The inhibition of migration and invasion of cancer cells by graphene via the impairment of mitochondrial respiration. Biomaterials 2014;35:1597–1607 24290441 10.1016/j.biomaterials.2013.11.020

[29] Ahmed SA, Gogal RM Jr., Walsh JE A new rapid and simple non-radioactive assay to monitor and determine the proliferation of lymphocytes: An alternative to [3H]thymidine incorporation assay. J Immunol Methods 1994;170:211–224 8157999 10.1016/0022-1759(94)90396-4

[30] Thomson RC, Yaszemski MJ, Powers JM, *et al.* Fabrication of biodegradable polymer scaffolds to engineer trabecular bone. J Biomater Sci Polym Ed 1996;7:23–38 10.1163/156856295x00805 7662615

[31] Williams JM, Adewunmi A, Schek RM, *et al.* Bone tissue engineering using polycaprolactone scaffolds fabricated via selective laser sintering. Biomaterials 2005;26:4817–4827 15763261 10.1016/j.biomaterials.2004.11.057

[32] Porter BD, Oldham JB, He LS, *et al.* Mechanical properties of a biodegradable bone regeneration scaffold. J Biomech Eng 2000;122:286–288 10923298 10.1115/1.429659

[33] Lotz JC, Gerhart TN, Hayes WC Mechanical properties of trabecular bone from the proximal femur: A quantitative CT study. J Comput Assist Tomogr 1990;14:107–114 2298972 10.1097/00004728-199001000-00020

